# Supplementary material for: Research on the degree of coupling of the Internet development level and agricultural——Ecological efficiency based on 2009–2018 data from 13 major grain-producing areas in China
Source: PLoS One. 2021 Jul 26;16(7):e0254078. doi: 10.1371/journal.pone.0254078 (PMC8312977; doi:10.1371/journal.pone.0254078)
Supplement: Attachment — Submitted filename: Reply to reviewer.docx [file pone.0254078.s004.docx]

**Reply to reviewer**

Dear editor and reviewer:

Hello！

Thank you very much for your recognition and support for our work. At the same time, your comments are of great help to the improvement of the quality of our articles. Thank you for your hard work and careful guidance.

The following is a response to the comments raised by the reviewing experts in combination with the revised article:

## 1. Reply to Reviewer #1's comments

First of all, thank the experts for the evaluation of our work：A good contribution.

### 1. Response to Comment 1:

Comment 1： Secondary data may have consistency issues such as data gaps for certain periods or regions. Did the author notice these issues while processing the historical information of different provinces and how did the author manage if there were such cases in the data frame?

Reply：We attach great importance to possible consistency issues in Secondary data ，and data consistency has an important impact on research，especially second-hand data is prone to fluctuations. The basic data used in this article are all from the statistical yearbooks and related reports issued by the Chinese government. Before using, this article compares the statistical yearbooks issued by the National Bureau of Statistics of China with the relevant data of the statistical yearbooks issued by the provincial statistical bureaus to ensure the authenticity of the data and the consistency of national and local statistics. According to the analysis of the relevant data, it can be seen that the basic data is relatively stable, which is mainly reflected in the steady increase in the Internet penetration rate in each production area. The basic data of agricultural eco-efficiency indicators have no significant fluctuations from year to year, and the data gaps between different regions are mainly derived from the realities of regional natural resources and economic and social development. Overall, the data consistency is better.

**Supplements corresponding to the article：**

In the revised article, a discussion on the issue of data consistency has been added. Its location is the first paragraph of 2.5 Potential restricted disposal in the revised article.

The supplementary content is：The consistency of the data has an important impact on the research. In particular, second-hand data is prone to fluctuations. The basic data used in this article were all obtained from statistical yearbooks and related reports issued by the Chinese government. Before using these data, the statistical yearbooks issued by the National Bureau of Statistics of China were compared with the relevant data from the statistical yearbooks issued by the provincial statistical bureaus to ensure the authenticity of the data and the consistency of the national and local statistics. According to the analysis of the relevant data, the basic data is relatively stable. This is mainly reflected in the steady growth of the Internet penetration rate in each production area, the basic data for the agricultural ecological efficiency index does not exhibit significant fluctuations in any year, and the data gap between the different regions is mainly from the result of the reality of the regional natural resources and the degrees of economic and social development. Overall, the data consistency is better.

### 2. Response to opinion 2:

Comment 2：It is said that qualitative data always supports quantitative ones in the field of social study. Does the author conduct some FGD or KII to verify the model output results by collecting feedback from the relevant stakeholders?

Reply：Focus group discussions (FGDs)、 Key informant interviews (KIIs) are an important method in the field of social research. Focus group discussions (FGDs) and Key informant interviews (KIIs) can provide support for quantitative research. In the process of research, we consulted some experts and scholars on the results of the measurement of agricultural ecological efficiency in the main grain producing areas to determine whether the measurement of agricultural ecological efficiency is accurate. In this process, we have further combined some existing research to support this part, which has been supplemented in the revised article. Of course, since the focus of this research is to analyze the coordination of agro-ecological efficiency and the development of the Internet, it mainly relies on official data for quantitative measurement. The further qualitative analysis of the research results will be the next stage of research work. For this reason, I am very grateful to the experts for giving us this opinion, which points out the direction for our next work.

**Supplements corresponding to the article：**

In the revised article, we added the support of expert opinions on the measurement results of agricultural eco-efficiency. Its position is in the middle of the first paragraph of the revised article 3.2. Measurement of agricultural ecological efficiency.

The supplementary content is： According to the results in Table 3, the results of this study are comparable to some existing research results [31, 36, 41], but the results are opposite to the conclusions of some experts. After comprehensive discussion, it was determined that the relevant results reflect the agricultural ecological efficiency of the main grain producing areas in China to a certain extent.

### 3. Reply to comments 3 and 4:

Comment3：Please insert the latitude and longitude value in the map data frame

Comment4：Please insert the province names on the map so that reader can compare the results among different provinces

Reply：According to the opinions of experts, the picture was revised in the revised article, adding the latitude and longitude, the name of the province, etc.

**Supplements corresponding to the article**：

In the revised article, we have revised all the pictures, adding the latitude and longitude, the name of the province and so on.

The supplementary picture is：

**Figure 1.**Main grain producing areas in China

**Figure 2.**Internet Development Level in Grain Main Areas

**4. Response to comment 5:**

Comment 5：It would be better if the author could summarize the socio-economic differences of different provinces in a tabular form.

Reply：The revised article supplements the socio-economic conditions of China's main grain producing areas in the form of a table, mainly including the regional GDP in 2018, the number of people at the end of the year, the gross agricultural production value, and the grain output.

**Supplements corresponding to the article**：

In the revised article, we have supplemented the socio-economic situation of China’s main grain producing areas, and its location is at the end of the revised article 2.1. Study area.

The supplementary content is：The economic and social conditions in China's 13 major grain producing areas in 2018, including the regional GDP, year-end population, gross agricultural production value, and grain output, are shown in Table 1.According to Table 1, Jiangsu Province has the highest GDP among the 13 main grain producing areas. Shandong Province is the most populous province among the 13 main grain producing areas, and it also has the highest agricultural production value. Heilongjiang Province has the highest grain output among the 13 main grain producing areas.

Table 1 Basic economic and social conditions in China's main grain producing areas

|  | Regional GDP (100 million yuan) | Population at the end of the year (10,000 people) | Agricultural production value (100 million yuan) | Grain production (10,000 tons) |
| --- | --- | --- | --- | --- |
| Hebei | 36010.27 | 7556.00 | 5707.00 | 3700.90 |
| Inner Mongolia | 17289.22 | 2534.00 | 2985.30 | 3553.30 |
| Liaoning | 25315.35 | 4359.00 | 4061.90 | 2192.40 |
| Jilin | 15074.62 | 2704.00 | 2184.30 | 3632.70 |
| Heilongjiang | 16361.62 | 3773.00 | 5624.30 | 7506.80 |
| Jiangsu | 92595.40 | 8051.00 | 7192.50 | 3660.30 |
| Anhui | 30006.82 | 6324.00 | 4672.70 | 4007.30 |
| Jiangxi | 21984.78 | 4648.00 | 3148.60 | 2190.70 |
| Shandong | 76469.67 | 10047.00 | 9397.40 | 5319.50 |
| Henan | 48055.86 | 9605.00 | 7757.90 | 6648.90 |
| Hubei | 39366.55 | 5917.00 | 6207.80 | 2839.50 |
| Hunan | 36425.78 | 6899.00 | 5361.60 | 3022.90 |
| Sichuan | 40678.13 | 8341.00 | 7195.60 | 3493.70 |

**5.Response to comment 6:**

Comment 6：Since uncertainty is interlinked in modeling, what are the potential areas of uncertainty in the model used in this study, and how did these uncertainties of model results address?

Reply：Research attaches great importance to the choice of model, the reason for model choice is：The selection of the correct model has an important impact on the research results. Based on the characteristics of the research many existing research results, the SBM model considering undesirable outputs was selected to measure the agricultural ecological efficiency and the degree of coupling coordination model was selected to calculate the degree of coordination. This model has been widely used in related research, and its advantages have been recognized by many scholars. The SBM model solves the problem that the measurement of the inefficiency in the radial model does not include relaxation variables, but during the application process, we must pay attention to the selection of its indicators. Therefore, based on existing research results, in this study, the reality in China was used to determine the relevant indicators in order to ensure the accuracy of the efficiency measurements. During the application of the degree of coupling coordination model, the α and β weighting for the determination of the coefficients has an important impact on the results. Therefore, in this study, several experts were consulted regarding the reality to determine the best coefficients in order to improve the scientific nature of the research.

**Supplements corresponding to the article**：

In the revised article, we have supplemented the reason for the selection of the model in this article, and its location is the second paragraph of 2.5 Potential restricted disposal of the revised article.

The supplementary content is：The selection of the correct model has an important impact on the research results. Based on the characteristics of the research many existing research results, the SBM model considering undesirable outputs was selected to measure the agricultural ecological efficiency and the degree of coupling coordination model was selected to calculate the degree of coordination. This model has been widely used in related research, and its advantages have been recognized by many scholars. The SBM model solves the problem that the measurement of the inefficiency in the radial model does not include relaxation variables, but during the application process, we must pay attention to the selection of its indicators. Therefore, based on existing research results, in this study, the reality in China was used to determine the relevant indicators in order to ensure the accuracy of the efficiency measurements. During the application of the degree of coupling coordination model, the α and β weighting for the determination of the coefficients has an important impact on the results. Therefore, in this study, several experts were consulted regarding the reality to determine the best coefficients in order to improve the scientific nature of the research.

### 6. Response to comment 7:

Comment 7：Potential limitations such as consistency issues of secondary data and the uncertainty of the model used are recommended to mention in the discussion or conclusion sections.

Reply：Regarding these issues raised by reviewers, we have already made supplements in the revised article. We believe that these limitations should be explained in more detail. It may not be obvious enough in conclusions and discussions. After comprehensively considering the layout of the article, We believe that the discussion of these issues should be placed before data analysis.

Supplements corresponding to the article：

In the revised article, we discussed the consistency of second-hand data and the uncertainty of the model used, and its location is 2.5 Potential restricted disposal of the revised article.

The supplementary content is：The consistency of the data has an important impact on the research. In particular, second-hand data is prone to fluctuations. The basic data used in this article were all obtained from statistical yearbooks and related reports issued by the Chinese government. Before using these data, the statistical yearbooks issued by the National Bureau of Statistics of China were compared with the relevant data from the statistical yearbooks issued by the provincial statistical bureaus to ensure the authenticity of the data and the consistency of the national and local statistics. According to the analysis of the relevant data, the basic data is relatively stable. This is mainly reflected in the steady growth of the Internet penetration rate in each production area, the basic data for the agricultural ecological efficiency index does not exhibit significant fluctuations in any year, and the data gap between the different regions is mainly from the result of the reality of the regional natural resources and the degrees of economic and social development. Overall, the data consistency is better.

The selection of the correct model has an important impact on the research results. Based on the characteristics of the research many existing research results, the SBM model considering undesirable outputs was selected to measure the agricultural ecological efficiency and the degree of coupling coordination model was selected to calculate the degree of coordination. This model has been widely used in related research, and its advantages have been recognized by many scholars. The SBM model solves the problem that the measurement of the inefficiency in the radial model does not include relaxation variables, but during the application process, we must pay attention to the selection of its indicators. Therefore, based on existing research results, in this study, the reality in China was used to determine the relevant indicators in order to ensure the accuracy of the efficiency measurements. During the application of the degree of coupling coordination model, the α and β weighting for the determination of the coefficients has an important impact on the results. Therefore, in this study, several experts were consulted regarding the reality to determine the best coefficients in order to improve the scientific nature of the research.

## Reply to Reviewer #2's comments

First of all, thank the experts for the evaluation of our work：The authors have selected an important topic and presented it in a better manner.

### 1. Response to comment 1:

Comment 1： Paper needs to be proofread by a native language speaker.

Reply：In order to improve the fluentness and readability of the article, we specially asked LetPub company to carry out a comprehensive polishing of the article. See the attachment for relevant certificates.

### 2. Response to comment 2:

Comment 2：Justifications of the methods selected are weak and needs to be improved in terms of explaining / indicating academic novelty in the paper.

Reply：The models used in the article are SBM-DEA and the coupling coordination degree model that considers undesired output. These two models have been recognized by many researches. The related development process and application situation have been explained in detail in the article. Considering the objectives and content of this research, these two models are reasonable to a certain extent.

Of course, with the development of metrology, various new models are constantly appearing, such as super-efficiency SBM models. In the next stage, the research team will combine the latest models to conduct further analysis on the research content. However, since the innovation of this research is mainly to take the lead in discussing the coordinated relationship between Internet development and agricultural ecological efficiency, it is also a more appropriate choice to apply the model recognized by the academic community at this stage. The article on the limitation of model application is further supplemented.

Supplements corresponding to the article：

In the revised article, we further analyzed the model selection problem, and its location is the second paragraph of 2.5 Potential restricted disposal of the revised article.

The supplementary content is：The selection of the correct model has an important impact on the research results. Based on the characteristics of the research many existing research results, the SBM model considering undesirable outputs was selected to measure the agricultural ecological efficiency and the degree of coupling coordination model was selected to calculate the degree of coordination. This model has been widely used in related research, and its advantages have been recognized by many scholars. The SBM model solves the problem that the measurement of the inefficiency in the radial model does not include relaxation variables, but during the application process, we must pay attention to the selection of its indicators. Therefore, based on existing research results, in this study, the reality in China was used to determine the relevant indicators in order to ensure the accuracy of the efficiency measurements. During the application of the degree of coupling coordination model, the α and β weighting for the determination of the coefficients has an important impact on the results. Therefore, in this study, several experts were consulted regarding the reality to determine the best coefficients in order to improve the scientific nature of the research.

### 3. Response to comment 3:

Comment 3： Discussion needs to be improved in terms of explaining the implication of the results in terms of both policy and academic contributions. Further, discuss the results in a comparative manner in order to explain where the results stand in academic discussions.

Reply：Based on the opinions of experts, combined with the research results, the discussion was further improved, the research results were compared with the existing research, and their academic contributions were analyzed.

Supplements corresponding to the article：

In the revised article, we further improved the discussion, and its location is the revised article 4 Conclusions and policy implications.

Supplementary content：(1) The Internet development level of China's major grain producing areas has consistently improved, and the average Internet penetration rate increased from 0.25 in 2008 to 0.54 in 2018. However, an information gap between the major grain producing areas still exists, and the Internet penetration rate in some areas needs to be further improved. Among them, the Internet penetration rate in the major grain producing areas around the Bohai Sea is higher, while that in Sichuan Province is lower.

(2) Previous studies have suggested that China's agricultural ecological efficiency is improving, but the overall efficiency has not yet reached the forefront, and there is a large gap between the different regions. The results of this study are consistent with previous research results, but most of the previous studies analyzed provinces and cities in China and did not specifically discuss the agricultural ecological efficiencies of the different grain production areas. This study further demonstrates the improvement of the agricultural ecological efficiency and the existence of regional gaps in the main grain producing areas in China. The average agricultural ecological efficiency of the 13 major grain producing areas in China increased from 0.45 in 2009 to 0.79 in 2018, reflecting the improvement of the overall agricultural ecological environment in the major grain producing areas in China. The agricultural ecological efficiency of the main grain producing areas in China is affected by their economic and natural conditions. Hebei, Liaoning, Jilin, Jiangsu, Shandong, Sichuan, and other regions have reached the forefront of agricultural ecological efficiency due to better natural conditions or more advanced technical support, while Anhui Province is restricted by its natural conditions and economic and technological development, resulting in a low agricultural ecological efficiency.

(3) Previous studies have not analyzed the relationship between the Internet development level and the agricultural ecological efficiency. In this study, an in-depth investigation of this relationship was conducted. The research results show that the degree of coupling and coordination between the Internet development level and the agricultural ecological efficiency in China’s main grain-producing areas continually increased from 2009 to 2018, showing that the interaction between the Internet development level and the agricultural ecological efficiency was continually strengthened, but the coordination between the two still has room for growth. The degree of coupling and coordination between the Internet development level and the agricultural ecological efficiency in China's main grain-producing areas increased from 0.58 in 2009 to 0.80 in 2018, reflecting the increasing influence of the interaction between the two. From the perspective of the different regions, the level of Internet development and the improvement of the agricultural ecological efficiency in the major grain-producing areas such as Liaoning, Jiangsu, and Hebei have achieved coordinated development, but Anhui Province has shortcomings.
